# Analysis of one-year follow-up results and treatment costs of patients with PTE in a tertiary care center

**DOI:** 10.5578/tt.20239607

**Published:** 2023-12-04

**Authors:** Ersin ERGÜL, Elif YILMAZEL UÇAR, Ömer ARAZ, Alperen AKSAKAL, Buğra KERGET, Leyla SAČLAM

**Affiliations:** 1 Department of Chest Diseases, Atatürk University Faculty of Medicine, Erzurum, Türkiye

## Abstract

**ABSTRACT**

**
Analysis of one-year follow-up results and treatment costs of patients with PTE in a tertiary care center
**

**Introduction:** *Pulmonary thromboembolism (PTE) is a life-threatening disease, with substantial treatment-related complications, difficult follow-up, treatment compliance, and high costs. This study aimed to assess treatment costs with various maintenance therapy regimens, complications, and patient adherence to treatment over a one-year follow-up period.
*

**Materials and Methods:** *This observational, prospective study included 142 patients with PTE who received maintenance anticoagulation therapy between November 2020 and March 2023. The patients were observed at three-month intervals for a year. Possible treatment-related complications, recurrence, mortality, and treatment costs were recorded.
*

**Results:** *Our results showed that there was no significant difference in bleeding risk based on the drugs used for initial or maintenance treatment. In mainte- nance therapy, low-molecular-weight heparin (LMWH), warfarin, and direct oral anticoagulant (DOAC) treatment regimens had similar treatment adher- ence and comparable efficacy and safety in terms of recurrence and bleeding (p> 0.05). Four patients (2.8%) were diagnosed with chronic thromboem- bolic disease. The one-year mortality rate was 24.6% (n= 35), of which 82.9% (n= 29) occurred within the first three months. Hospital mortality rates with the different maintenance therapies were 8.8% in the LMWH group, 5.7% in the warfarin group, and 3.2% in the DOAC group. The annual cost of using LMWH was higher than that of rivaroxaban, apixaban, and warfarin (p< 0.001) while there was no significant cost difference between DOACs and warfarin (p> 0.05).
*

**Conclusion:** *In our study, the LMWH, warfarin, and DOAC treatment regimens had similar efficacy, safety, and patient compliance. In terms of cost, LMWH was the costliest while DOAC and warfarin were similar.
*

**Key words:** *Pulmonary thromboembolism; anticoagulant drugs; bleeding; recurrence; cost
*

**ÖZ**

**
Üçüncü basamak bir merkezde PTE’li hastaların bir yıllık takip sonuçları ve tedavi maliyetlerinin analizi
**

**Giriş:** *Pulmoner tromboemboli (PTE); tedaviye bağlı ciddi komplikasyonlar gelişebilen, takibi ve tedavi uyumu zor olan, ciddi mali- yete sahip önemli bir morbidite ve mortalite nedenidir. Bu çalışmanın amacı çeşitli tedavi rejimlerinde hastaların tedaviye uyumunun, tedaviye bağlı gelişebilecek komplikasyonların değerlendirilmesi ve tedavi maliyetlerinin belirlenmesidir.
*

**Materyal ve Metod:** *Bu prospektif, gözlemsel çalışmaya Kasım 2020-Mart 2023 tarihleri arasında PTE tanısı alan ve idame antikoagü- lan tedavi planlanan 142 hasta dahil edildi. Hastalar üçer ay aralıklarla bir yıl boyunca takip edildi. Tedaviye bağlı gelişebilecek komplikasyonlar, nüks, mortalite ve tedavi maliyetleri kaydedildi.
*

**Bulgular:** *Araştırma sonuçlarımız başlangıçta ve idame tedavide kullanılan ilaçlar ile kanama güvenilirliği arasında anlamlı fark olma- dığını göstermiştir. İdame tedavide de DMAH, varfarin ve DOAK tedavi rejimlerinin tedavi uyumu, rekürrens ve kanama yönünden benzer etkinlik ve güvenilirliğe sahip olduğunu göstermiştir (nüks için p= 1,000, p= 1,000, p= 1,000 sırası ile, kanama için p= 1,000, p= 1,000, p= 0,577 sırası ile). Dört hastaya (%2,8) kronik tromboembolik hastalık (KTEH) tanısı konmuştur. Bir yıllık mortalite ora- nımız %24,6 (35 hasta) olup, %82,8’i (29 hasta) ilk üç ay içerisindeydi. İdame tedavilerin hastane mortalite oranları; DMAH verilen grupta %8,8, varfarin grubunda %5,7, DOAK grubunda ise %3,2 olarak izlendi. Maliyet değerlendirilmesinde DMAH kullanmanın yıllık maliyetinin rivaroksaban, apiksaban ve varfarin kullanımından daha yüksek olduğunu (p< 0,001), DOAK’lar ile varfarin arasın- da ise anlamlı fark olmadığını göstermiştir (p= 1,000).
*

**Sonuç:** *Çalışmamızda DMAH, varfarin ve DOAK tedavi rejimlerinin etkinlik ve güvenilirlikleri benzer bulunmuştur. Maliyet yönünden DMAH’ler yüksek maliyete sahip olup DOAK ve varfarin maliyetleri ise benzerdir.
*

**Anahtar kelimeler:** *Pulmoner tromboemboli; antikoagülan ilaçlar; kanama; nüks; maliyet
*

## INTRODUCTION

Pulmonary thromboembolism (PTE) is an acute cardiovascular disease with high mortality and morbidity (1). Early and effective treatment of PTE is very important in the prevention of mortality and morbidity that it may cause (1). There are various treatment options for PTE, including anticoagulation alone, catheter-directed thrombolysis, full-dose systemic thrombolysis, reduced-dose systemic thrombolysis, catheter embolectomy, surgical embolectomy, and mechanical circulatory support devices (2).

PTE is traditionally considered an acute disease, but individuals who survive the acute stage may develop recurrent PTE and deep vein thrombosis episodes, bleeding complications related to anticoagulant therapy, cardiovascular events, and rarely complications such as chronic thromboembolic pulmonary hypertension (CTEPH) in the long-term natural course of the disease (3). Although patients hospitalized for acute PTE are provided appropriate care and follow-up, there is currently no clear algorithm for outpatient follow-up after acute PTE (2).

PTE also poses a heavy economic burden on healthcare services, and according to a study conducted in the USA, hospitalization costs associated with PTE treatment averaged $8.764 per patient, while another study showed an average cost of

$16.644 per patient over 12-month follow-up (4,5).

In a study conducted in our country in which patients were followed up for an average of 200 days, the hospitalization cost per patient was calculated as

$717 (6).

Our aim in this study was to determine treatment compliance, treatment-related complications, recurrence, and mortality rates, investigate the prevalence of CTEPH, and to determine the treatment and follow-up costs of patients with acute PTE in the first year after discharge.

### MATERIALS and METHODS

**Study Design**

This prospective, observational, single-center study included 142 patients who were diagnosed with PTE between November 1, 2020 and March 1, 2022 underwent inpatient treatment in the Chest Diseases Clinic of Atatürk University Faculty of Medicine Research Hospital, and were followed up in our outpatient clinic after discharge. The institutional review board approved the study protocol (B.30.3.ATA.0.23.00/49). Before starting the study, all patients were informed about the study and their written consent was obtained.

### Study Population and Procedures

Patients over the age of 18 years who were diagnosed with PTE and received maintenance anticoagulant therapy between November 2020 and March 2023 were included in the study. Patients who did not

undergo computed tomography pulmonary angiography (CTPA) and echocardiographic (ECHO) examinations at the time of diagnosis or did not volunteer to participate in the study were excluded.

The patients’ demographic characteristics (age, sex), time from symptom onset to hospital admission, presence of recurrence at admission, pulmonary complaints, Wells and simplified pulmonary embolism severity index (sPESI) scores, thrombus location determined by CTPA at diagnosis (main pulmonary artery, lobar branch, segmental, subsegmental branch), PTE risk scoring, presence of predisposing factors, right ventricular (RV) dysfunction on ECHO at the time of diagnosis, the treatment started after diagnosis and whether thrombolytic therapy was given, the anticoagulant used as maintenance therapy [low-molecular-weight heparin (LMWH), warfarin, direct oral anticoagulant (DOAC)], treatment-related complications (minor/major hemorrhage), hemorrhage at three, six, nine, and 12 months, recurrence, mortality rates, and costs were recorded. Patients who did not come for follow-up were interviewed by phone call.

No special groups were formed in the planning of maintenance therapy for our patients. In general, patients with malignancy were discharged with LMWH. Patients who had no contraindications or problems related to transportation to the health center and could attend international normalized ratio (INR) follow-up were discharged with warfarin. Patients who were not considered good candidates for warfarin (had transportation problems to the health center, said they could not attend INR follow- up, and/or may confuse the warfarin dose because of the need for dose adjustment) and could afford the additional pharmacy fees were discharged with LMWH or DOAC.

### Definitions

Treatment adherence; was defined as the degree to which the patient took the prescribed medication as prescribed (at the correct time, frequency, and dose).

Major hemorrhage; was defined as bleeding from the brain or other critical organ that caused hemoglobin levels to fall by 2 g/dL or more, required two or more red cell transfusions to treat, and was severe enough to cause death (7).

Minor hemorrhage; was defined as a small amount of bleeding or simple anemia in the absence of signs of

blood loss that was managed with minimal interventions such as observation, ice, and compression without discontinuing anticoagulant therapy, which considering the short half-life of these drugs would pose a potential thrombosis risk (7).

Recurrence; was defined as the reoccurrence of PTE in a patient previously diagnosed as having PTE, with or without treatment.

CTEPH was diagnosed based on the following criteria: persistent dyspnea or functional limitation despite taking regular anticoagulants for at least three months; perfusion defects on V/Q scintigraphy; increased pulmonary artery pressure on ECHO; and mean pulmonary artery pressure ≥20 mmHg on right heart catheterization (RHC) (8).

Mortality that occurred within the first 30 days after hospitalization for PTE was classified as hospital mortality and analyzed separately from mortality that occurred in the following 11 months.

Cost was evaluated as all expenses (including accommodation, transportation, and food costs at the time of admission to the hospital and the amounts paid to the pharmacy) including post-discharge expenses (laboratory tests, imaging tests, and extra INR tests and associated examination fees for those followed up with warfarin). Social insurance expenditures and cost to the patients for the drugs used for maintenance therapy during follow-up, as well as costs at three, six, nine, and 12 months and the total annual cost were added into the analysis.

### Statistical Analysis

Statistical analysis was performed using IBM SPSS Statistics version 22.0 software program for Windows. Descriptive statistics for quantitative variables were expressed as mean, standard deviation, median, and range (minimum-maximum) values. Descriptive statistics for qualitative variables were expressed as frequency and percentage values. In the normality analysis using the Shapiro-Wilk test, the quantitative data did not show normal distribution. Therefore, nonparametric tests were used for analyses. Mann- Whitney U test, Kruskal-Wallis H test, and Spearman’s correlation analysis were used to determine the relationships between the parameters. Chi-square test was used in the pairwise analysis of qualitative data. The results were evaluated at 95% confidence interval and p< 0.05 was considered significant.

## RESULTS

One hundred and forty-two PTE patients included in the study had a mean age of 66 ± 16 years, 56% of them were aged 65 years or older, and 59% (n= 84) were females. At the time of admission, 13 patients (9.2%) had a history of previous PTE and were classified as recurrent. The most common presenting complaint was dyspnea (79%). The patients’ demographic and clinical findings at admission and the treatments received are summarized in Table 1.

When treatment-related complications were evaluated, 11 patients (7.7%) developed major

hemorrhage and 25 (17.6%) developed minor hemorrhage during hospitalization (Table 2). There was no statistically significant relationship between the patients’ bleeding complications and initial treatment received (Table 2).

Complications that occurred during patient follow- up are summarized in Table 3. Mortality was particularly high in the first three months (n= 29). A total of 35 patients died (24.6%). Eight of these deaths (5.6%) occurred within the first 30 days and were classified as hospital mortality (Table 3).

Findings consistent with CTEPH were observed in eight patients (5.6%). Two of these patients did not consent to the RHC procedure. In the other six patients, mean pulmonary artery pressure was ≤20 mmHg on RHC, and none of the patients were diagnosed with CTEPH. These patients remain under follow-up for chronic thromboembolic disease (CTED).

When our patients attended outpatient clinic follow- up and were asked whether they used their medications, all reported using their medications at the prescribed time, frequency, and dose and were evaluated as having high treatment compliance.

There was no statistically significant relationship between the patients’ bleeding complications during follow-up and the maintenance therapy they received (p> 0.05) (Table 4).

The recurrence rate during treatment was 2.8%. When patients who developed recurrence during follow-up were compared in terms of comorbidities, three of the patients had no comorbidities. A higher recurrence rate was observed in patients with cerebrovascular accident and malignancy. Seven of the patients with recurrence had two or more comorbidities. However,

**Table:** 

**Table 1.** Baseline characteristics of the patients (n= 142) (continue)		
	**Number**	**Percent (%)**
CAD	23	16
**Number of comorbidities**		
1	47	33
≥2	61	43
0	34	24
**RV dysfunction on echocardiography**	109	76.8
**Embolism location on CTPA**		
Main pulmonary artery	77	54.6
Lobar artery	106	75.2
Segmental artery	126	89.4
Subsegmental artery	60	42.6
**Initial treatment**		
Thrombolytic + LMWH	53	37.3
Enoxaparin sodium	88	62.0
Fondaparinux	1	0.7
**Maintenance therapy**		
LMWH	45	32
Warfarin	35	25
DOAC	62	44
DVT: Deep vein thrombosis, PTE: Pulmonary thromboembolism, sPESI: Simplified pulmonary embolism severity index, COPD: Chronic obstructive pulmonary disease, CHF: Chronic heart failure, CVD: Cerebrovascular disease, CAD: Coronary arterial dis- eases, OSAS: Obstructive sleep apnea syndrome, RV: Right ventricu- lar, CTPA: Computed tomography pulmonary angiography, LMWH: Low-molecular-weight heparin, DOAC: Direct oral antico- agulants.

comparative analysis of recurrence and comorbidities revealed no significant relationship (p= 0.242). There was also no statistically significant relationship between recurrence and maintenance therapy (p> 0.05) (Table 4).

Characteristics of the nonsurviving patients are summarized in Table 5. Mean age of the nonsurvivors was 77 ± 11 years, and 30 (86%) were over 65 years old. All of the patients who died had at least one risk factor. Immobilization was the most common risk factor, followed by malignancy. Hospital mortality rates for the different maintenance therapies were 8.8% in the LMWH group, 5.7% in the warfarin group, and 3.2% in the DOAC group.

Figure 1 shows the social insurance expenditures and cost to the patients for the drugs used for maintenance therapy during follow-up, as well as costs at three, six, nine, and 12 months and the total annual cost. For all treatment regimens, costs were highest at three months and lowest at 12 months. The total annual cost associated with patient follow-up and treatment were 31.370 ± 1523 Turkish lira (TL) for LMWH, 3895 ± 1254 TL for warfarin, 3723 ± 601 TL

for rivaroxaban, and 3933 ± 1597 TL for apixaban.

There was a significant relationship between maintenance drugs and cost (p< 0.001). There was no significant difference between DOACs and warfarin (p> 0.05), whereas LMWH therapy had significantly higher cost at three, six, nine, and 12 months and total annual cost compared to rivaroxaban, apixaban, and warfarin (p< 0.001).

## DISCUSSION

In this follow-up study, we evaluated patients with PTE every three months for the first year after discharge. Our results showed that there was no significant difference in bleeding risk according to the drugs used in initial or maintenance treatment. LMWH, warfarin, and DOAC maintenance therapy regimens had similar treatment adherence and comparable efficacy and safety in terms of recurrence and bleeding. In the cost assessment, the use of LMWH was associated with higher annual cost than rivaroxaban, apixaban, and warfarin while there was no significant difference in cost between DOACs and warfarin.

**Table:** 

**Table 2.** Comparison of hemorrhage with initial treatment					
	**n**	**Major hemorrhage, n (%)**	**p**	**Minor hemorrhage, n (%)**	**p**
Thrombolytic + LMWH	53	7 (13)	0.101	12 (23)	0.224
LMWH	89	4 (4)	0.101	13 (15)	0.224
LMWH: Low-molecular-weight heparin.					

**Table:** 

**Table 3.** Complications and mortality rates during follow-up				
	**0-3 months, n (%)**	**3-6 months, n (%)**	**6-9 months, n (%)**	**9-12 months, n (%)**
Hemorrhage	1 (0.9)	0	1 (1)	3 (3.1)
Recurrence	2 (1.9)	1 (1)	1 (1)	0
Mortality	29 (20.4)	1 (1)	3 (2.6)	2 (1.8)
CTEPH	0	0	0	0
CTED	0	2 (1.4)	6 (4)	0
CTEPH: Chronic thromboembolic pulmonary hypertension, CTED: Chronic thromboembolic disease.				

**Table:** 

**Table 4.** Comparison of hemorrhage and recurrence complications at follow-up with the maintenance therapy									
	**n**	**Month 3, n (%)**	**p**	**Month 6, n (%)**	**p**	**Month 9, n (%)**	**p**	**Month 12, n (%)**	**p**
**Hemorrhage**									
Warfarin	35	0	1	-		0	1	1 (3.0)	1
LMWH	45	1 (2.2)	0.42	-		0	1	1 (2.5)	1
DOAC	62	0	1	-		1 (1.7)	0.42	1 (1.8)	0.57
**Recurrence**									
Warfarin	35	0	1	0	1	0	1	-	
LMWH	45	2 (4.4)	0.17	0	1	0	1	-	
DOAC	62	0	1	1 (1.6)	1	1 (1.7)	1	-	
LMWH: Low-molecular-weight heparin, DOAC: Direct oral anticoagulants.

PTE is a condition that develops acutely but in the long term can persist and even result in death due to complications associated with both the disease itself and the treatments given. As the disease is especially common in the older population, it is often accompanied by comorbidities and polypharmacy, and treatment compliance is essential. The duration of anticoagulation therapy in PTE patients is at least three months and may vary depending on their risk factors. Bleeding is the main side effect of the drugs used to treat the disease. Therefore, patients should be monitored closely for drug-related complications.

In meta-analysis studies evaluating the risk of bleeding, it has been observed that thrombolytic therapy initially increased the risk of major hemorrhage and this risk was higher in advanced age (>65 years) (9,10). In our study, although 64% of the patients receiving thrombolytic therapy were older, no significant relationship was found between thrombolytic therapy and bleeding risk. This may be related to the prescription of low-dose (50 mg) thrombolytic therapy in our clinic to older patients and high bleeding risk based on the results of previous studies (11-13). In their study evaluating the

link between bleeding risk and maintenance therapy, Wysokinski et al. have prospectively assessed rates of major and minor hemorrhage at six months in 750 patients treated with apixaban (n= 224), rivaroxaban (n= 163), or enoxaparin (n= 363) and observed no significant difference (14). Uppuluri et al. have also found no significant difference in bleeding rates in their retrospective evaluation of 131 cancer patients who received DOAC, LMWH, or warfarin therapy

(15). In a meta-analysis published by Li et al. in 2019, DOACs were associated with higher bleeding rates than LMWH, contrary to our study (16). This difference was shown to be a result of poor treatment adherence and therefore shorter duration of use with parenteral LMWH therapy compared to oral DOACs among the patients included in the study. Two recently published cohort studies, one of which compared patients receiving apixaban and warfarin and the other comparing patients receiving apixaban, rivaroxaban, dabigatran, and warfarin, have demonstrated less bleeding in patients receiving DOACs (17,18). In these studies, the difference between DOACs and warfarin has been attributed to the initial concomitant administration of LMWH and warfarin in conventional

**Table:** 

**Table 5.** Characteristics of nonsurviving patients compared to all patients		
	**Nonsurvivors (n= 35)**	**Total patients (n= 142)**
Age (years), mean ± SD	77 ± 11	66 ± 16
Sex, female	19 (54.2%)	84 (40.8%)
**Risk factors**		
Immobilization	15 (42.8%)	36 (25.4%)
Malignancy	10 (28.6%)	21 (14.8%)
Obesity	7 (21.0%)	29 (20.4%)
**Number of risk factors**		
1	6 (17.1%)	30 (21.1%)
≥2	29 (82.9%)	102 (71.9%)
**Comorbidities**		
Cardiac diseases	11 (31.5%)	109 (76.7%)
Neurological diseases	9 (25.7%)	18 (12.7%)
Pulmonary diseases	7 (20.0%)	21 (14.7%)
**Number of comorbidities**		
1	14 (40.0%)	47 (33.1%)
≥2	21 (60.0%)	61 (43.0%)
**Clinical severity**		
Massive	7 (20.0%)	22 (15.5%)
Submassive high risk	22 (62.8%)	72 (50.7%)
Submassive low risk	6 (17.2%)	32 (22.5%)
**Complications**		
Major hemorrhage	5 (14.2%)	11 (7.7%)
Minor hemorrhage	10 (28.5%)	25 (17.6%)
**Maintenance therapy**		
LMWH	20 (57.1%)	45 (31.6%)
DOAC	12 (34.2%)	62 (43.6%)
Warfarin	3 (8.7%)	35 (24.6%)
LMWH: Low-molecular-weight heparin, DOAC: Direct oral anticoagulants.

therapy (bridging therapy) and patients’ noncompliance with INR follow-up.

In our study, we observed that the different drug groups used for maintenance therapy were similarly effective in preventing recurrences of VTE. A recent meta-analysis of 2226 patients and a retrospective cohort study with 446 patients both demonstrated no significant differences in VTE recurrence between patients receiving DOAC and LMWH (19,20). In a data analysis study by Kohen et al. including 14.086 patients receiving apixaban, LMWH, or warfarin,

similar recurrence rates were observed in patients receiving warfarin and LMWH, while a lower rate of recurrence was observed in the apixaban arms (21), which was attributed to the initiation of LMWH in high-risk patients and DOACs in lower-risk patients. In a retrospective six-month observational cohort study published by Weycker et al. in 2020, a total of 55.641 patients receiving apixaban and warfarin were compared and a lower recurrence rate was observed in patients using apixaban (22). The inability to optimally administer warfarin was noted as the reason for this.

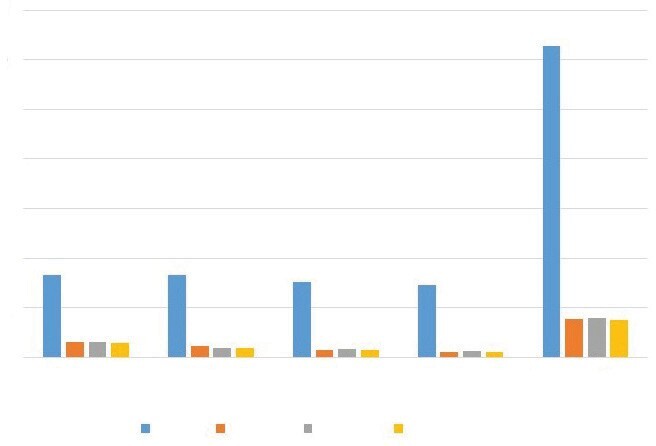

**Figure 1.** Comparison of treatment and follow-up costs by drugs.

PTE can cause up to 62% mortality depending on the patient’s clinical picture at the time of admission (8). Today, PESI score is a useful tool for determining the mortality risk of PTE patients (23). Dentali et al. have retrospectively evaluated 538 PTE patients and reported an overall mortality rate of 23.2% at three months, 30.2% at six months, and 37.1% at 12 months. They have stated that PESI scores provided accurate predictions in patients without cancer (23). Justyna et al. have prospectively followed 402 PTE patients for two years and reported that age was important in determining mortality after discharge

(24). In our study, 70% of the patients had a high PESI score and the one-year mortality rate was 24.6%. Most of the nonsurviving patients were older, had risk factors such as immobilization, malignancy, and obesity, and had at least one comorbidity. Among the comorbidities, cardiac pathologies were most common, followed by neurological pathologies. This indicates that mortality in our patients was not only associated with PTE but other factors. When we look at the hospital mortality rates with different maintenance therapies, this rate was found to be 6.7% in the LMWH group in a previous study conducted in our clinic and similarly was 8.8% in the LMWH group in the present study (12). The DOAC group had the lowest hospital mortality rate. The higher mortality in the LMWH group may be due to the fact that this treatment is prescribed to higher-risk patients.

PTE imposes a heavy economic burden on health services (4,5). The present study also examined the one-year costs of different treatment strategies and showed that using LMWH was much costlier than using rivaroxaban, apixaban, and warfarin. There was no significant difference in cost between DOACs and warfarin. Coşkuner et al. have evaluated the six-month costs of different treatment regimens and reported that LMWH cost more than warfarin and DOAC. Contrary to our study, warfarin has been reported to have lower costs than DOACs. This may be due to the inability to calculate the nonpharmacological costs associated with warfarin use (25). Türk et al. have retrospectively compared the costs of PTE maintenance therapy and determined that DOACs were more affordable than warfarin (6). However, this study sought to answer the question of what the costs would have been for 118 patients treated with warfarin if they had been given alternative treatments. In other words, it was conducted using a model and was not based on actual patient data for DOACs and LMWH. However, while drug, outpatient appointments, and transportation costs were included in the cost of warfarin therapy, only drug costs were included for DOAC and LMWH therapy when calculating the costs in the maintenance period based on this model. By recording nonpharmacological expenditures in the maintenance period and including prospective follow-up data, our study presents more objective data.

This study has certain limitations. First of all, data were collected during the patients’ outpatient follow- up visits, which were affected by the onset of the COVID-19 pandemic and pandemic-related restrictions in place at the start the study. Patients who could not come to the outpatient clinic for such reasons were each called and interviewed. For patients in different cities, laboratory and imaging tests were requested in the cities where they were located, and the results were evaluated through the national health records system (e-Nabız). Secondly, we evaluated the patients’ treatment compliance based on self-reported medication use when they came to the outpatient clinic for follow-up. For those who could not attend outpatient visits, we attempted to make this assessment when they were called by phone, based on statements from themselves and their relatives, as well as by reviewing their electronic prescriptions through the national centralized prescription system (Medula) and the e-Nabız system.

## CONCLUSION

The results of this study showed that there was no significant difference in bleeding risk according to the drugs used in initial and maintenance treatment. The maintenance therapy regimens of LMWH, warfarin, and DOAC showed similar efficacy and safety in terms of treatment adherence, recurrence, and bleeding. The one-year treatment and follow-up costs of maintenance therapy were highest with LMWH, while there was no significant difference between DOACs and warfarin. These results may support the potential use of DOACs as a first-line option for the treatment of PTE in eligible patients.

**Ethical Committee Approval:** This study was approved by Atatürk University Faculty of Medicine Clinical Research Ethics Committee (Decision no: 49, Date: 30.03.2023).

### CONFLICT of INTEREST

The authors declare that they have no conflict of interest.

## AUTHORSHIP CONTRIBUTIONS

Concept/Design: EE, EYU Analysis/Interpretation: BK, AA, EE Data acqusition: EE, OA

Writing: EE, EYU

Clinical Revision: OA, LS

Final Approval: EYU, OA, LS, AA

## REFERENCES

1. *
  Tarbox AK, Swaroop M. Pulmonary embolism. Int J Crit Illn Inj Sci 2013; 3(1): 69-72. https://doi. org/10.4103/2229-5151.109427*
2. *
  Rivera-Lebron B, McDaniel M, Ahrar K, Alrifai A, Dudzinski DM, Fanola C, et al. Diagnosis, treatment and follow up of acute pulmonary embolism: Consensus practice from the PERT Consortium. Clin Appl Thromb Hemost 2019; 25: 1076029619853037. https://doi. org/10.1177/1076029619853037*
3. *
  Klok FA, Cohn DM, Middeldorp S, Scharloo M, Büller HR, van Kralingen KW, et al. Quality of life after pulmonary embolism: Validation of the PEmb-QoL Questionnaire. J Thromb Haemost 2010; 8(3): 523-32. https://doi. org/10.1111/j.1538-7836.2009.03726.x*
4. *
  Fanikos J, Rao A, Seger AC, Carter D, Piazza G, Goldhaber SZ. Hospital costs of acute pulmonary embolism. Am J Med 2013; 126(2): 127-32. https://doi.org/10.1016/j. amjmed.2012.07.025*
5. *
  Spyropoulos AC, Lin J. Direct medical costs of venous thromboembolism and subsequent hospital readmission rates: An administrative claims analysis from 30 managed care organizations. J Manag Care Pharm 2007; 13(6): 475-86. https://doi.org/10.18553/jmcp.2007.13.6.475*
6. *
  Turk M, Aldag Y, Oguzulgen I, Ekim N. A cost comparison of warfarin vs enoxaparine or new oral anticoagulants used for the treatment of patients with pulmonary embo- lism. Tuberk Toraks 2016; 64(3): 198-205. https://doi. org/10.5578/tt.24153*
7. *
  Garcia DA, Crowther M, Moreira ME. Management of bleeding in patients receiving direct oral anticoagulants. U: UpToDate, Post TW ur UpToDate [Internet] Waltham, MA: UpToDate. 2018.
  *
8. *
  Arseven O, Bingöl Z, Çöplü L, Erol S, Oğuzülgen İ, Okumuş N, et al. Türk Toraks Derneği Pulmoner Tromboembolizm Tanı ve Tedavi Uzlaşı Raporu. 2021.
  *
9. *
  Chatterjee S, Chakraborty A, Weinberg I, Kadakia M, Wilensky RL, Sardar P, et al. Thrombolysis for pulmonary embolism and risk of all-cause mortality, major bleeding, and intracranial hemorrhage: A meta-analysis. JAMA 2014; 311(23): 2414-21. https://doi.org/10.1001/ jama.2014.5990*
10. *
  Marti C, John G, Konstantinides S, Combescure C, Sanchez O, Lankeit M, et al. Systemic thrombolytic ther- apy for acute pulmonary embolism: A systematic review and meta-analysis. Eur Heart J 2015; 36(10): 605-14. https://doi.org/10.1093/eurheartj/ehu218*
11. *
  Yilmazel Ucar E, Araz O, Kerget B, Yilmaz N, Akgun M, Saglam L. Comparison of long‐term outcomes of 50 and 100 mg rt‐PA in the management of acute pulmonary thromboembolism. Clin Respir J 2018; 12(4): 1628-34. https://doi.org/10.1111/crj.12721*
12. *
  Ucar EY, Akgun M, Araz O, Tas H, Kerget B, Meral M, et al. Comparison of LMWH versus UFH for hemorrhage and hospital mortality in the treatment of acute massive pul- monary thromboembolism after thrombolytic treatment: Randomized controlled parallel group study. Lung 2015; 193(1): 121-7. https://doi.org/10.1007/s00408-014-*

*9660-z*

13. *
  Ucar EY, Araz O, Akgun M, Meral M, Kalkan F, Saglam L, et al. Low-molecular-weight heparin use with thromboly- sis: Is it effective and safe? Ten years’ clinical experience. Respiration 2013; 86(4): 318-23. https://doi. org/10.1159/000346203*
14. *
  Wysokinski WE, Houghton DE, Casanegra AI, Vlazny DT, Bott‐Kitslaar DM, Froehling DA, et al. Comparison of apix- aban to rivaroxaban and enoxaparin in acute cancer‐asso- ciated venous thromboembolism. Am J Hematol 2019; 94(11): 1185-92. https://doi.org/10.1002/ajh.25604*
15. *
  Uppuluri EM, Burke KR, Haaf CM, Shapiro NL. Assessment of venous thromboembolism treatment in patients with cancer on low molecular weight heparin, warfarin, and the direct oral anticoagulants. J Oncol Pharm Pract 2019; 25(2): 261-8. https://doi. org/10.1177/1078155217730129*
16. *
  Li A, Garcia DA, Lyman GH, Carrier M. Direct oral antico- agulant (DOAC) versus low-molecular-weight heparin (LMWH) for treatment of cancer associated thrombosis (CAT): A systematic review and meta-analysis. Thromb Res 2019; 173: 158-63. https://doi.org/10.1016/j.throm- res.2018.02.144*
17. *
  Cohen AT, Sah J, Dhamane AD, Hines DM, Lee T, Rosenblatt L, et al. Effectiveness and safety of apixaban vs warfarin in patients with venous thromboembolism with risk factors for bleeding or for recurrences. Adv Ther 2023; 40(12): 5447-63. https://doi.org/10.1007/s12325-*

*023-02440-1*

18. *
  Lawal OD, Aronow HD, Shobayo F, Hume AL, Taveira TH, Matson KL, et al. Comparative effectiveness and safety of direct oral anticoagulants and warfarin in patients with atrial fibrillation and chronic liver disease: A nationwide cohort study. Circulation 2023; 147(10): 782-94. https:// doi.org/10.1161/CIRCULATIONAHA.122.060687*
19. *
  Rungjirajittranon T, Owattanapanich W, Chinthammitr Y, Ruchutrakool T, Suwanawiboon B. Direct oral anticoagu- lants versus low-molecular-weight heparins for the treat- ment of acute venous thromboembolism in patients with gastrointestinal cancer: A systematic review and meta-analysis. Thromb J 2022; 20(1): 41. https://doi. org/10.1186/s12959-022-00399-7*
20. *
  Song Y, Yang D, Hou D, She J, Song Y. Comparison of the efficacy and safety of rivaroxaban and low-molecu- lar-weight heparin in Chinese lung cancer patients with nonhigh-risk pulmonary embolism. Thromb Journal 2023; 21(1): 1-14. https://doi.org/10.1186/s12959-023-*

*00453-y*

21. *
  Cohen A, Keshishian A, Lee T, Wygant G, Rosenblatt L, Hlavacek P, et al. Effectiveness and safety of apixaban, low-molecular-weight heparin, and warfarin among venous thromboembolism patients with active cancer: A US claims data analysis. Thromb Haemost 2021; 121(3): 383-95. https://doi.org/10.1055/s-0040-1718728*
22. *
  Weycker D, Wygant GD, Guo JD, Lee T, Luo X, Rosenblatt L, et al. Bleeding and recurrent VTE with apixaban vs war- farin as outpatient treatment: Time-course and subgroup analyses. Blood Advances 2020; 4(2): 432-9. https://doi. org/10.1182/bloodadvances.2019001081*
23. *
  Dentali F, Riva N, Turato S, Grazioli S, Squizzato A, Steidl L, et al. Pulmonary embolism severity index accurately predicts long-term mortality rate in patients hospitalized for acute pulmonary embolism J Thromb Haemost 2013; 11(12): 2103. https://doi.org/10.1111/jth.12420*
24. *
  Justyna A, Dzikowska-Diduch O, Pacho S, Ciurzynski M, Skowronska M, Wyzgal-Chojecka A, et al. Decreased hae- moglobin level measured at admission predicts long term mortality after the first episode of acute pulmonary embo- lism. J Clin Med 2022; 11(23): 7100. https://doi. org/10.3390/jcm11237100*
25. *
  Coşkuner MB, Öztuna F, Bülbül Y, Özsu S. The cost of pulmonary thromboembolism treatment. Tuberk Toraks 2020; 68(3): 293-304. https://doi.org/10.5578/tt.70104*

